# Event and Comment

**Published:** 1908-06-15

**Authors:** 


### DENTAL REGISTER.

Vol. LXII. June 15, 1908. No. 6.

### EVENT AND COMMENT.

### The Dental Convention.

This is the season of all the year in which the dental society or convention seems to flourish most luxuriantly. It would seem that the long winter months are not altogether used as a hibernating period, but for deep thinking and experimenting that the spring and summer of professional activity may bloom with new ideas and technical advances. When one reads the multitude of announcements of dental meetings, and sees the programs filled with names of eminent men as speakers and essayist and the scores of clinics on all manner of things new and old, he wonders if the game will ever be played out. There seems at present little likelihood that the convention idea may become extinct in the minds of people in all walks of social, political, or professional activity, as there probably never was a time when as many conventions were being held, or so many people were attending them. One is inclined to question the value of so much effort in time and means, and to inquire if the same end could not more economically be attained by the writing and publishing of tracts, essays, books, and by the more general dissemination of this knowledge through the periodical literature of the profession.

The amount of money spent annually by dentists in attending conventions would pay for a great many subscriptions to our dollar journals, but would the man who bought a dental periodical be very much interested in the men who contribute to its pages, whom he had never seen, in reading it? The facts as nearly as we are able to observe them are that few dentists comparatively contribute to our general literary output, and only a slightly larger number contribute to the technical department of our professional advance. We have also observed that in local state and national conventions there are certain men who attend and do things with considerable regularity, while others participate or attend as suits their convenience or inclinations. We have also observed that the larger percentage of practitioners scarcely ever attend a dental convention of any sort. Now why do some always go to the dental society and others never? Is it a matter of habit or education? We believe that both reasons will apply with great probability in certain cases. There are certain dentists that you expect to meet at dental conventions as certainly as that there will be a convention held. They have the habit and would be dreadfully uncomfortable should they miss a meeting; and, while it is a habit with them, it is one that has been cultivated or is the result of an enlightened conception of duty or a high appreciation of the educational value and pleasure to be obtained from such gatherings. Why some don't go at all is a more difficult question to answer. We might say that such men are ignorant of the pleasure or benefits to be had, or have no idea of their professional obligations, and let it go at that. But this kind of a statement would never make a convert of such a man and would be resented by all such persons.

Most men who enter our profession start with the one wrong conception that it is a good profession to have because it will yield a comfortable and permanent income or support. They qualify on this basis and take up their work as a tradesman or mechanic would do, and have no other notion than that it is up to them to make all the money they can, with little or no thought that they have entered an educated profession where there is laid upon them the most sacred obligations to render to the public humane services of the highest value, and that the most valuable service will many times afford slight return in money. In other words they were not called into the profession and never having heard the call to come up higher, they do not feel the need of counseling or associating with those who have learned that professional men have responsibilities and moral obligations that many other occupations may avoid.

A professional man, and we speak here more especially of a dentist, can not be ignorant of those things which qualify him to serve his patients with a more or less complete knowledge of the science and art of his profession, the more complete his knowledge the more valuable his service. Now how can a dentist keep posted as to what is materializing in his profession if he neither reads books, nor associates with men of his own profession in social or association work? We believe it is absolutely necessary that one should if practicable come into personal contact with the great leaders of thought and action in our profession to get the necessary inspiration to become even moderately useful. It is true that one can by diligent reading and study in the solitude of one's own fireside dig out the essentials of all that is done in professional progress, but it will require a high order of devotion and grit to accomplish much without the stimulus that always results from personal contact, even should it be by the senses of sight and hearing alone. One can not see another in action without mentally forming in his own mind some notion of the sort of man he is or estimating in technical values his utterances. If these be contrary to ones beliefs or practices he either discredits the man or his utterances, or what is better one begins a process of overhauling his own ideas or practices to determine more definitely their true values: and it is just this process of mental action which leads, when intelligently guided, to the development of a desire to know more, and inspires one with a desire to at least keep up with the advances others are making, if not to excell. This means education in its broadest and best sense, and is one if not the chief value of associated work.

We can then truly say that Dental Conventions both small and great have a most useful function and should not be ignored but fostered and utilized by all for mutual inspiration and benefit, for it is here that men usually find out what they need. If one is self satisfied or conceited he is likely to meet others who may be able to show him that there are other ways of arriving at satisfactory solutions of the many mysterious problems that he has not thought of, and he is made more conservative or judicial in his methods or pronouncements. When one discovers his need he rarely fails to set himself the task of supplying it, he takes his work more seriously, and by and by, he develops a love for his work, because he does it well and makes continued progress, which not only satisfies his own desires but pleases his clients.

A whole chapter on the great friendships that have resulted through the working together of men in associated effort might be written, but let it suffice to say, that many times where men have contended strenuously with each other for their ideas, and have fought the matters out in open meetings where they have had the combined judgment of many to assist them to right conclusions, have through such intercourses become ardent admirers and friends because they have become acquainted with the spirit which inspired each other.

We can certainly affirm that no dentist has become eminent, or even well known, who has persistently absented himself from dental associations and has confined his works to the limits of his practice or even to the publication of the results of his practical observations or research. On the contrary all our great men in both science and art have constantly contributed the great bulk of their work through the dental societies with which they are affiliated, therefore as a matter of precedent we can cordially indorse the dental society and commend it conscientiously to any who may have little idea of its worth.

We sometimes find dentists who do not affiliate with societies because of some personal prejudice or pique, because they think they cannot harmonize with some people who are members of the society, or may be because they can't approve of the way the organization conducts its affairs. Such personal prejudices are of course likely to be unfounded in fact, and will soon disappear when one falls into harmony with the management and its methods of doing business.

There are none too many dental societies, we need more of them, and every ethical practitioner should connect himself with some society and undertake to do what he can for its best interests. When one does this in a consecrated and loyal manner, his development is certain and his occupation will cease to be distasteful to him. He will delight in his work because he does it well, and he becomes conscious that he is serving his fellow man to the best of his ability, and what may be a comfort to some he will soon find that his patients will note the difference in his attitude and reward him with their confidence as never before.

